# Synthetic lethality of MCL-1 inhibition and CAR-T therapy in aggressive B-cell lymphoma

**DOI:** 10.1038/s41375-026-02884-8

**Published:** 2026-02-12

**Authors:** Jing Gao, Xiaohong Zhao, Qing Yin, Allen Hu, Kevin Qiu, Loryn Blackburn, Lenny Lei, Rui Xiong, Chengfeng Bi, Jeffrey W. Craig, Craig A. Portell, Marco L. Davila, Michael E. Williams, Jianguo Tao

**Affiliations:** 1 Department of Pathology, University of Virginia Comprehensive Cancer Center, Charlottesville, VA USA; 2 Department of Malignant Hematology, H. Lee Moffitt Cancer Center & Research Institute, Tampa, FL USA; 3 Department of Bioinformatics, University of California San Diego, La Jolla, CA USA; 4 Division of Hematology and Oncology, University of Nebraska Medical Center, Omaha, NE USA; 5 Division of Hematology and Oncology, University of Virginia Comprehensive Cancer Center, Charlottesville, VA USA; 6 Department of Medicine, Roswell Park Comprehensive Cancer Center, Buffalo, NY USA

**Keywords:** B-cell lymphoma, Cancer therapeutic resistance

## Abstract

Aggressive B-cell lymphomas, driven by MYC overexpression, exhibit rapid progression, resistance to therapies, and poor survival. While chimeric antigen receptor (CAR)-engineered T cells have demonstrated remarkable clinical efficacy in B-cell lymphomas, nearly half of patients who initially respond to CAR-T therapy eventually develop resistance and disease progression. In this study, we report the presence of residual drug-tolerant persister (DTP) and resistant lymphoma cells remaining within a highly immunogenic tumor microenvironment (TME) induced by the MCL-1 inhibitor (MCL-1i) S63845. MCL-1 inhibition downregulates MYC and activates the STAT1-interferon inflammatory pathway, promoting cytotoxic T-cell infiltration with reduced tumor-associated myeloid cells both in vitro and in vivo. Sublethal dose of the MCL-1i enhances TME immunogenicity and reawakens anti-tumor immune responses in murine models. We show that MCL-1i and CD19-targeted CAR-T cells reciprocally overcome resistance to each single-agent therapy, and combining MCL-1i with CD19 CAR-T cells significantly improves treatment efficacy, resulting in near-complete eradication of MYC-driven lymphoma in vivo. Together, these findings highlight a synergistic, dual-pronged therapeutic strategy targeting both tumor-intrinsic survival pathways and the immunosuppressive TME. This combinatorial one-two-punch approach offers a promising path to eliminate DTP and residual disease, prevent relapse and pave the way for deep clinical remissions in aggressive B-cell lymphomas.

## Introduction

Aggressive B-cell lymphomas are frequently driven by MYC overexpression and are clinically characterized by rapid progression, poor therapeutic response, and short overall survival. Despite advances in targeted therapy and immunotherapy, including the development of CAR-T therapy, treatment resistance remains the major barrier to curative outcomes in these malignancies. CAR-T therapy has demonstrated remarkable efficacy in patients with B-cell lymphomas [1–3]. However, approximately 50% of those who initially respond ultimately relapse [4, 5]. MYC, a pleiotropic transcription factor, regulates genes critical for cell growth, cell cycle progression, survival and apoptosis. MYC deregulation, via chromosomal translocation, amplification, or transcriptional activation, is common in aggressive B-cell lymphomas [6]. While Burkitt lymphoma and high-grade B-cell lymphoma with MYC and BCL2 rearrangements characteristically harbor MYC gene rearrangement, many other aggressive lymphoma subtypes, such as diffuse large B-cell lymphoma (DLBCL) and mantle cell lymphoma (MCL), exhibit elevated MYC levels despite lacking rearrangements. High MYC expression often correlates with poor prognosis in these lymphomas [7, 8]. MYC overexpression promotes DNA replication and cellular proliferation but also induces pro-apoptotic stress via TP53 activation [9, 10], thus requiring cooperative mechanisms to sustain tumor progression. Additionally, MYC fosters an immunosuppressive TME by suppressing MHC Class II expression [11], disrupting cytotoxic T-cell and NK cell surveillance [12], recruiting tumor-associated macrophages [13–15], upregulating immunosuppressive molecules CD47 and PD-L1 [16], and promoting the exclusion of T and NK cells [13].

MCL is a clinically and biologically heterogeneous subtype of non-Hodgkin lymphoma, with most patients having aggressive clinical presentations, while a subset has a more indolent course [17]. It is characterized by the hallmark t(11;14)(q13;q32) translocation, leading to cyclin D1 overexpression [18]. Additionally, disrupted DNA damage response pathways through ATM or TP53 mutations play important roles in MCL pathogenesis. ATM loss occurs in ~40% of newly diagnosed MCL patients, allowing the cells with unrepaired DNA to escape from p53 surveillance [18]. Further, MYC protein expression is a high-risk factor in MCL; concurrent MYC overexpression and TP53 alterations confer a dismal prognosis with a median overall survival of less than 3 years [19]. Clinically, significant therapeutic progress and improved survival have been realized for MCL patients. Bortezomib-based therapy for newly diagnosed MCL such as VR-CAP (R-CHOP regimen, but with bortezomib substituted for vincristine) was shown more effective than R-CHOP but with additional, predominantly hematologic toxicity [20]. The era of targeted therapeutics for MCL began with the BTK inhibitor ibrutinib approval, based upon impressive single-agent activity in relapsed and refractory MCL [21]. Frontline chemoimmunotherapy with bendamustine plus rituximab (BR) has emerged as a standard of care in recent years, with recent clinical trials incorporating BTKi with the BR backbone Ibrutinib and significantly improved progression-free survival (PFS) in older and previously untreated MCL patients, albeit without significant improvement in overall survival [22]. Acalabrutinib, a second-generation BTKi, was also added to the BR backbone for front-line MCL therapy and showed significantly improved PFS, a trend toward improved OS, and fewer toxicities than observed with the ibrutinib study [23]. TP53-mutant MCL typically is associated with a more aggressive clinical course and poor survival following standard chemoimmunotherapy regimens. To address this unmet need for these high-risk patients, a multicenter, phase 2 study of zanubrutinib, obinutuzumab, and venetoclax (BOVen) in untreated patients with MCL with a TP53 mutation was performed by Kumar and colleagues [24]. This study showed that nearly all patients responded and achieved a complete response, with some patients in deep remission by imaging and undetectable measurable residual disease being able to discontinue therapy [24]. These promising results support future evaluation of this regimen, along with other strategies to deploy bispecific T-cell-engaging monoclonal antibodies or CAR-T therapy as post-induction consolidation to further deepen therapeutic response and prolong remission duration for patients with high-risk MCL. Despite this progress, a cure remains elusive for most patients, making it imperative to better understand mechanisms of therapeutic resistance that can be targeted therapeutically.

Myeloid cell leukemia-1 (MCL-1) encodes an anti-apoptotic BCL-2 family protein that inhibits cytochrome c release by binding pro-apoptotic proteins, including BID and BIM. MCL-1 overexpression is implicated in the development of B-cell lymphomas [25, 26], and MCL-1 cooperates with MYC to promote lymphomagenesis [27, 28] by counteracting MYC-induced apoptosis. It is, therefore, of considerable therapeutic interest to inhibit the expression and/or function of MCL-1 in aggressive B-cell lymphomas reliant on MYC overexpression. Our prior studies identified MCL-1 dependency as a novel vulnerability in MYC-associated B cell lymphomas, making MCL-1 inhibition (MCL-1i) a promising therapeutic strategy [29, 30]. However, previous clinical studies and our own pre-clinical data have shown that treatment with single-agent anti-BCL-2 family member therapy, such as BCL-2 or MCL-1 inhibitors, is associated with the rapid acquisition of resistance.

The successes of immunotherapy have shifted the paradigm of cancer treatment, especially with the advent of CD19-directed CAR-T cell therapy for the treatment of B-cell lymphomas [31, 32]. Although CD19 CAR-T cell therapy provides durable remission for many patients, those with MYC overexpression often show resistance to these agents, leaving them with limited options [33, 34]. Potential mechanisms hampering CAR-T therapy efficacy include T cell exclusion/exhaustion, intrinsic genetic and epigenetic dysregulation in lymphoma cells, and the extrinsic TME [35, 36]. Thus, there is an urgent need to develop effective therapeutic strategies to ultimately overcome CAR-T resistance and prevent the fatal progression of lymphoma.

Given MYC’s central role in immune evasion and lymphoma progression, we investigated the interplay between MYC and MCL-1 and the immune responses elicited by MCL-1i. We found that MCL-1 inhibitor treatment eliminates most lymphoma cells, but that DTP cells emerged, characterized by MYC suppression and STAT1-type I interferon (IFN) axis activation. Single-cell and bulk transcriptome analysis, combined with immunogenomic profiling ex vivo and in vivo, revealed that MCL-1 inhibition downregulates MYC, activates the STAT1-IFN cascade, enhances cytotoxic T-cell activity, and reduces tumor-associated myeloid cells (TAMs), thereby reinvigorating anti-tumor immunogenicity and providing the opportunity for CAR-T therapy to yield deeper and longer-lasting tumor suppression.

## Methods

### Cell lines

MCL cell lines HBL-2, Mino, Maver-1 and Z138, DLBCL cell line DHL-16 and Burkitt lymphoma (BL) cell line Namalwa were purchased from ATCC. These cells and their S63845-resistant derivatives were cultured in RPMI-1640 (Gibco-Invitrogen) with10% FBS, penicillin (100 U/mL) and streptomycin (100 μg/mL) and maintained at 37 °C in 5% CO_2_. The P493-6 B lymphoblastoid cell line, with tetracycline-inducible MYC expression, was maintained with Tet system-approved FBS. Cell lines were routinely tested for mycoplasma using the Universal Mycoplasma Detection Kit (Fisher Scientific, CAT# MP093050301).

### Statistics

Unless otherwise stated, comparisons between two groups were performed using a two-tailed Student’s t-test. Data are presented as mean ± SD from at least 3 independent experiments. One-way analysis of variance (ANOVA; multiple-group comparison) or log-rank (Mantel-Cox) test (survival study) was used to determine statistical significance by GraphPad Prism software. *P* values of less than 0.05 were considered statistically significant. *P < 0.05, **P < 0.01, ***P < 0.005, ****P < 0.001.

## Results

### MCL-1 is constitutively expressed and serves as a key regulator of cell survival in lymphoma cells

**Fig. 1:**
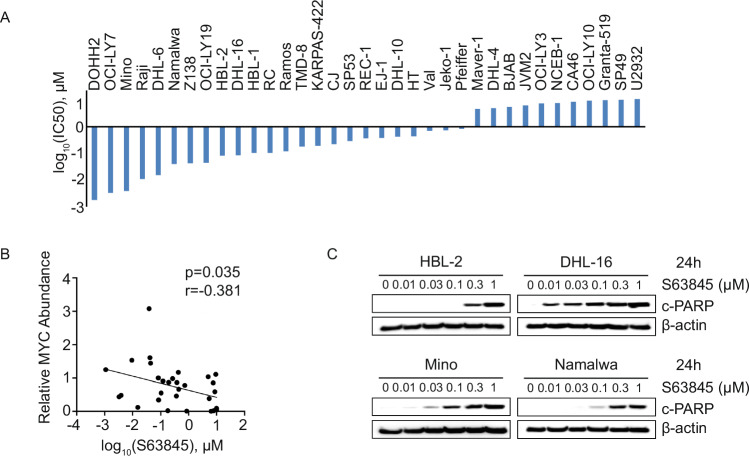
Aggressive B-cell lymphoma cell lines are sensitive to MCL-1 inhibition. **A** Bar plot showing the log_10_-transformed 50% inhibitory concentrations (IC50) of B-cell lymphoma cell lines treated with S63845 for 72 h. **B** Correlation analysis between MYC protein expression levels and Log-transformed S63845 IC_50_ values. IC_50_ values were derived from MTT assay, and protein levels were determined by western blotting. Band intensities were quantified and normalized to HBL-2 as the reference cell line. **C** Western blot analysis of cleaved PARP (c-PARP) in lymphoma cell lines treated with the indicated concentrations of S63845 for 24 h indicating apoptosis induction. Data in (**A**) are representative of at least three independent experiments.

MYC-associated lymphomas, which include BL and aggressive forms of double-hit lymphoma (DHL), DLBCL and MCL, are characterized by a high proliferative capacity and strong anti-apoptotic signaling. To evaluate whether these lymphomas depend on MCL-1 for survival, we treated a panel of MYC-associated B-cell lymphoma cell lines with the selective MCL-1i S63845. As shown in Fig. 1A and Supplementary Fig. 1A, most MYC-associated lymphoma lines were highly sensitive to S63845. Western blot analysis confirmed elevated levels of both MCL-1 and MYC proteins (Supplementary Fig. 1B, MCL-1 and β-actin protein abundance by western blot was previously published in our earlier work [37]) in lymphoma cell lines, consistent with prior reports [29, 30]. Furthermore, correlation analysis between MYC protein levels and S63845 sensitivity (IC50) revealed a negative relationship, indicating that higher MYC expression is associated with increased sensitivity to MCL-1 inhibition (Fig. 1B). In line with this, S63845 treatment induced marked apoptotic cleavage of PARP in all four tested MYC-associated lymphoma lines (Fig. 1C). Collectively, these findings highlight MCL-1i as a novel therapeutic vulnerability for MYC-driven aggressive lymphoma.

### MCL-1i induced DTP and resistant lymphoma cells are characterized by MYC downregulation and STAT1 upregulation and confer antitumor immunity

**Fig. 2:**
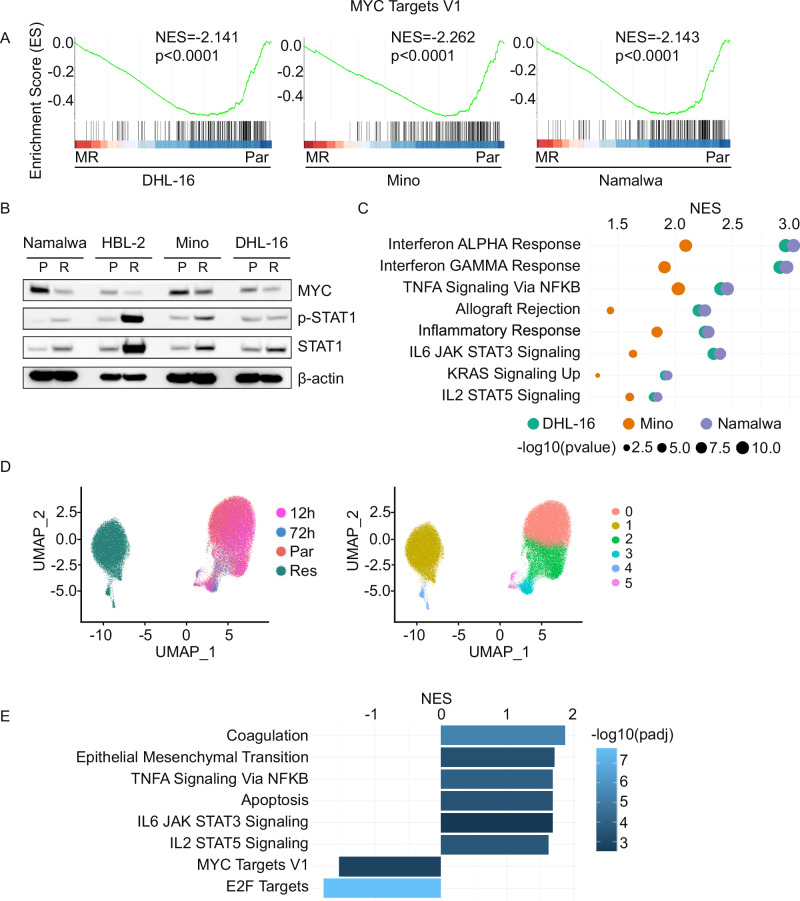
Alterations in MYC and STAT1 signaling pathways during the development of MCL-1i resistance. **A** GSEA showing negative enrichment of the MYC Targets V1 gene set in S63845-resistant (MR) cell lines compared to parental (Par) lines. NES, normalized enrichment score. **B** Western blot analysis showing decreased MYC and increased phosphorylated STAT1 (p-STAT1) and total STAT1 levels in S63845-resistant (R) cell lines compared to parental (P) lines**. C** Shared positively enriched HALLMARK pathways of S63845-resistant (MR) cell lines relative to parental (Par) lines, as determined by GSEA. NES, normalized enrichment score. **D** Uniform Manifold Approximation and Projection (UMAP) visualization showing 6 distinct transcriptional subpopulations in scRNA-seq data of untreated Namalwa cells (Par), treated Namalwa cells collected at 12 h (12 h) and 72 h (72 h) of S63845 exposure, and resistant cells derived from chronic S63845 exposure (Res)**. E** Enrichment analysis by Enrichr for upregulated and downregulated pathways based on DEGs in S63845-resistant versus parental Namalwa cells.

Although MCL-1i demonstrates potent efficacy against MYC-driven aggressive lymphomas [29, 30], mono-targeting MCL-1 poses a clinical challenge due to eventual progression driven by therapy resistance. It is increasingly recognized that such resistance arises through the rapid emergence and transient survival of small, viable DTP cell populations [38]. These DTP cells, along with fully resistant tumor subclones, frequently appear after an initial therapeutic response [39, 40]. To develop rational combination strategies that can sustain MCL-1i efficacy, we investigated the temporal evolution of resistance and its underlying molecular mechanisms. We generated four MCL-1i resistant cell lines through chronic exposure to MCL-1i using gradually escalating doses of MCL-1i for 8–12 weeks (Supplementary Fig. 2A), as well as DTP cells via short-term (72 h, IC_50_ dose) treatment with S63845 in MYC-driven lymphoma cell lines (HBL-2 [MCL], DHL-16 [DLCBL], Mino [MCL], Namalwa [BL]). We performed bulk RNA-seq and western blot analyses on parental, resistant and DTP cells, as well as on primary lymphoma samples. As shown in Fig. 2A–C and Supplementary Fig. 2B, C, transcriptomic profiling revealed consistent downregulation of MYC and upregulation of STAT1 in S63845-resistant compared to parental cell lines. Gene set enrichment analysis (GSEA) further demonstrated activation of type-I interferon, JAK/STAT and inflammatory pathways, along with suppression of MYC signaling.

Next, we explored transcriptomic dynamics following MCL-1i using an in vitro system designed to identify gene signatures and pathways associated with cell survival during S63845 treatment. We established four distinct cell states using the aggressive B-cell lymphoma cell line Namalwa: (1) untreated parental cells, (2) cells treated with S63845 (at IC50) for 12 h, (3) DTP cells after 72 h treatment with S63845 (at IC50), and (4) cells that developed resistance after chronic exposure to S63845. To characterize treatment-induced molecular evolution, we performed single-cell RNA sequencing (scRNA-seq) across these four states. To identify subpopulations and signaling pathways associated with MCL-1i resistance, we focused on the difference between parental and chronically treated cells. Cell populations were clustered and visualized using uniform manifold approximation and projection (UMAP). We determined the adaptive transcriptome reprogramming after the chronic MCL-1i treatment. As shown in Fig. 2D, UMAP revealed six transcriptionally distinct clusters (clusters 0-5) across the treatment conditions (right panel). Differentially expressed gene analysis revealed distinct transcriptomic subpopulations among parental, DTP (72 h) and resistant cells. GSEA indicated that MYC target genes were enriched in the parental population but downregulated in chronically treated cells (Fig. 2E). In contrast, NF-κB and Interferon signaling pathways were enriched in MCL-1i-treated cells. Specifically, clusters 1, 4 were predominantly associated with cells treated chronically (Supplementary Fig. 2D). To further assess the functional relevance of these transcriptional states, AUCell analysis showed positive enrichment of IFN-alpha and IFN-gamma signaling pathways, while MYC target genes were negatively enriched in MCL-1i-treated clusters (Supplementary Fig. 2E).

**Fig. 3:**
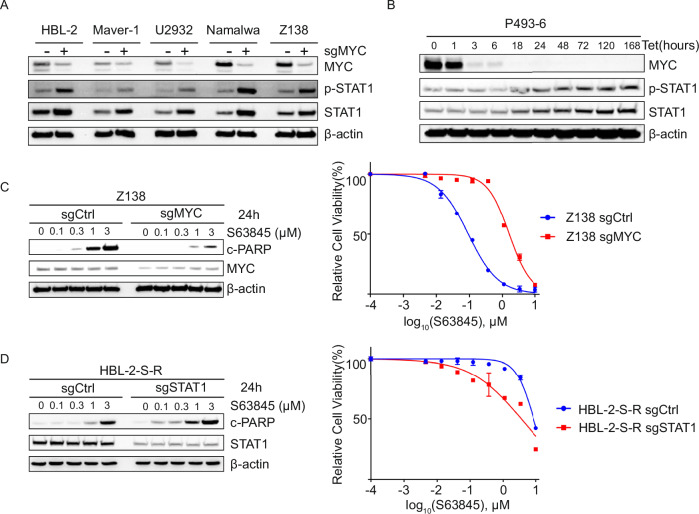
MCL-1i-induced MYC downregulation and STAT1 upregulation contribute to the development of resistance to MCL-1 inhibition. **A** Western blot analysis showing decreased MYC and increased phosphorylated STAT1 (p-STAT1) and total STAT1 levels in MYC knockdown (+) cell lines compared to parental controls (–)**. B** Western blot showing increased p-STAT1 and total STAT1 levels in MYC-off P493-6 cells by 1 μM doxycycline treatment. **C** Western blot analysis of cleaved PARP (c-PARP) and MYC protein expression in parental (sgCtrl) and MYC knockdown (sgMYC) cell lines treated with the indicated concentrations of S63845 for 24 h (left). Cell viability assay demonstrating enhanced proliferation of MYC knockdown cells (Z138) following S63845 treatment compared to parental cells (right). **D** Western blot analysis of cleaved PARP (c-PARP) and STAT1 expression in S63845-resistant cell lines (sgCtrl) and corresponding STAT1 knockdown cell lines (sgSTAT1) treated with the indicated doses of S63845 for 24 h (left). Cell viability assay showing reduced proliferation in STAT1 knockdown S63845-resistant cell lines (HBL-2, right). Data shown in (**C**, **D**) are representative of at least three independent experiments.

To further investigate the functional roles of MYC and STAT1 in mediating MCL-1i resistance, we performed CRISPR/Cas9-mediated MYC knockdown in a panel of MYC-driven lymphoma cell lines, including MCL, DLBCL and BL. Sustained MYC knockdown for over 3 days resulted in increased expression and phosphorylation of STAT1 protein (Fig. 3A). Similarly, when we modulated MYC expression by a tetracycline-regulated P493-6 human lymphoblastoid cell line, we observed an inverse correlation between MYC and STAT1 protein levels (Fig. 3B). These results indicated the repressive role of MYC in STAT1 expression and activation in lymphoma. As shown in Fig. 3C and Supplementary Fig. 3A, B, MYC knockdown led to a reduction in MCL-1i-induced lymphoma cell apoptosis, as evidenced by decreased PARP cleavage in western blot and reduced cell death in viability assays. Conversely, CRISPR/Cas9-mediated knockdown of STAT1 enhanced MCL-1i-induced apoptosis, as measured by both PARP cleavage and cell viability (Fig. 3D, Supplementary Fig. 3C, D). These findings suggest that MCL-1i-induced MYC downregulation and STAT1 upregulation contribute to the development of resistance to MCL-1 inhibition.

### MCL-1i treatment-induced MYC downregulation contributes to STAT1 upregulation and is associated with Type I IFN and inflammatory signaling pathway activation

**Fig. 4:**
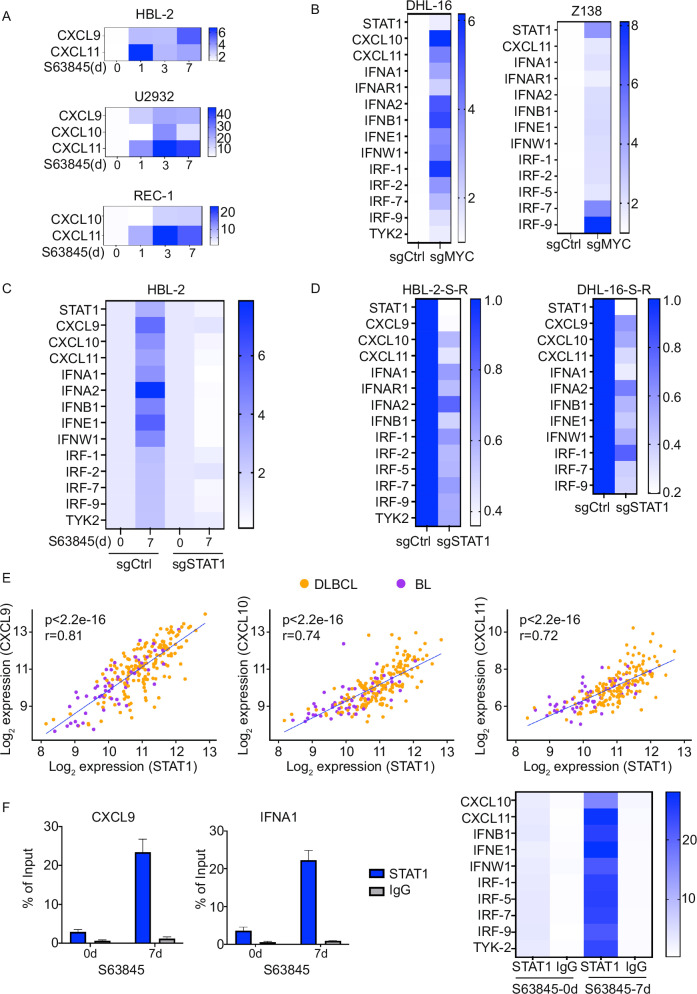
MYC downregulation induced by S63845 treatment leads to STAT1 upregulation and activation of Type I IFN signaling pathways. **A** Heatmap showing increasing expression of CXCL9, CXCL10 and CXCL11 in parental B-cell lymphoma cell lines (HBL-2, U2932 and REC-1) treated with the IC50 doses of S63845 at different time points as indicated. **B** Heatmap showing elevated levels of STAT1, CXCL10, CXCL11, and genes associated with IFN and inflammation in MYC knockdown DHL-16 and Z138 cells compared to parental cell lines**. C** Heatmap showing elevated levels of STAT1, CXCL9, CXCL10, CXCL11, and genes associated with IFN and inflammation in HBL-2 parental and STAT1 knockdown cells treated with the IC50 doses of S63845 for 7 days**. D** Heatmap showing elevated levels of STAT1, CXCL9, CXCL10, CXCL11, and genes associated with IFN and inflammation in STAT1 knockdown HBL-2 and DHL-16 S63845 resistance cells**. E** Correlation plots showing positive association between STAT1 mRNA levels and CXCL9, CXCL10, CXCL11 mRNA levels in aggressive B-cell lymphoma patient samples (DLBCL, orange; BL, purple). **F** ChIP using STAT1-specific antibody showing STAT1 enrichment at the promoters of genes involved in CXCL9, CXCL10, CXCL11 and genes associated with IFN and inflammation.

To dissect the regulatory role of MYC in IFN signaling and its upstream modulator STAT1 during the development of MCL-1i resistance, we examined the effect of MYC downregulation on immune signaling by assessing transcript levels of IFN and inflammatory pathway-related genes. Real-time quantitative PCR (qRT-PCR) analyses showed that acute MCL-1i treatment (1 to 7 days) induced a type I IFN and inflammatory response (Supplementary Fig. 4A), including elevated expression of chemokines such as CXCL9, CXCL10 and CXCL11 (Fig. 4A). Notably, MYC knockdown via sgRNA further amplified the expression of these inflammatory genes (Fig. 4B and Supplementary Fig. 4B), consistent with enhanced STAT1 activation observed in western blot assays. To determine whether transcriptional activation requires STAT1, we performed the STAT1 knockdown using sgRNA. STAT1 depletion significantly attenuated IFN/inflammatory gene induction in both parental MYC-driven lymphoma cells upon MCL-1i treatment and MCL-1i-resistant cell lines (Fig. 4C, D and Supplementary Fig. 4C). Consistent with these functional findings, we analyzed transcriptomic data from publicly available MYC-driven human lymphoma datasets [41]. Correlation analysis revealed strong associations between MYC or STAT1 expression and that of inflammatory chemokines, including CXCL9, CXCL10 and CXCL11 (Fig. 4E, Supplementary Fig. 4D) and IFN response genes (Supplementary Fig. 4E), supporting the clinical relevance of this regulatory axis. To investigate the direct regulatory role of STAT1 on IFN/inflammatory-signature genes, we performed chromatin immunoprecipitation (ChIP) using a STAT1-specific antibody. As shown in Fig. 4F, STAT1 was significantly enriched at the promoters of genes involved in type I IFN signaling, inflammation, and T-cell chemotaxis, including key chemokines. This enrichment was associated with enhanced sensitivity to MCL-1i, suggesting that STAT1-mediated transcription contributes to overcoming resistance in MCL-1i-resistant lymphoma cells. Importantly, we found that upregulation of the type I IFN pathway, downregulation of MYC, and activation of the STAT1-CXCL9/10/11 axis occurred not only in cells with acquired MCL-1i resistance but also in DTP cells. These findings indicate that both acute and chronic MCL-1i can elicit lymphoma-intrinsic anti-tumor immunogenicity through STAT1-mediated inflammatory responses.

### MCL-1i treatment reinvigorates the TME toward immunogenicity in syngeneic MYC-driven lymphoma in vivo

**Fig. 5:**
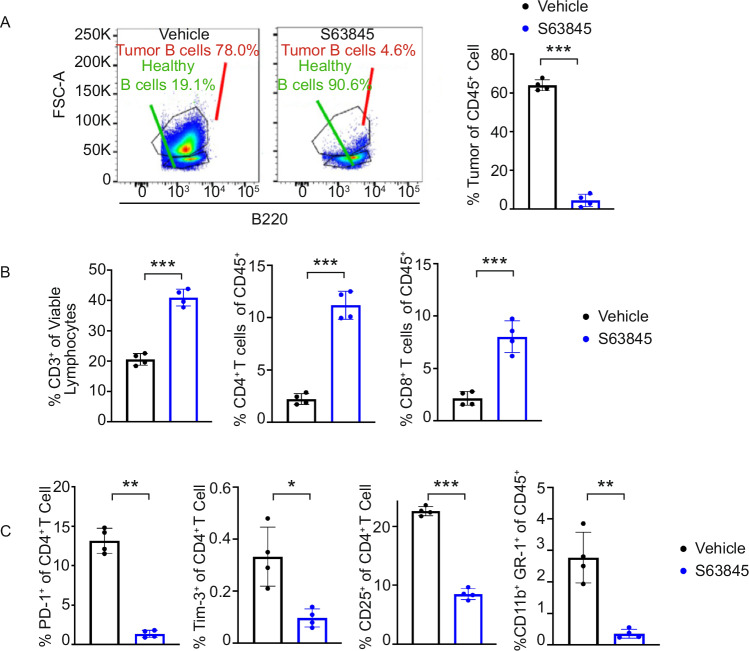
MCL-1 inhibition with S63845 reinvigorates TME towards immunogenicity in a syngeneic MYC-driven lymphoma mouse model. **A** Representative flow cytometry dot plot (left) and quantification (right) showing a reduction in tumor B cells among CD45^+^ cells following S63845 treatment. **B** Increased proportions of CD3^+^ T cells among viable lymphocytes, and CD4^+^ and CD8^+^ T cells among CD45^+^ cells after S63845 treatment measured by flow cytometry. **C** Decreased proportions of PD-1^+^, Tim-3^+^, and CD25^+^ cells among CD4^+^ T cells, as well as GR-1^+ ^CD11b^+^ myeloid cells among CD45^+^ cells following S63845 treatment, measured by flow cytometry.

Given that MCL-1i treatment leads to MYC downregulation and activation of STAT1-mediated type I IFN and inflammatory signaling, we investigated whether MCL-1i treatment could promote anti-tumor immunity in vivo. To this end, we treated syngeneic Eμ-MYC lymphoma-bearing mice, a model of MYC-driven aggressive lymphoma, with a sublethal dose of S63845 (25 mg/kg, weekly) as shown in Supplementary Fig. 5A. Mice were sacrificed, and immune profiling was performed on the residual tumor burden. Spleens were harvested and analyzed via multicolor flow cytometry to assess changes in tumor-infiltrating lymphocytes. Flow cytometry revealed that S63845 treatment selectively reduced the number of lymphoma cells while sparing normal splenic B lymphocytes (Fig. 5A). Further, compared to vehicle-treated controls, S63845 significantly increased splenic infiltration by CD3^+^ T-cells, including both CD4^+^ and CD8^+^ T-cell subsets (Fig. 5B). Furthermore, MCL-1i treatment reduced the frequency of immunosuppressive cell populations, including CD25^+^ CD4^+^ regulatory T cells (Tregs), PD-1^+^ /Tim-3^+^ exhausted T-cells, and CD11b^+^ GR-1^+^ myeloid-derived suppressor cells (MDSCs) (Fig. 5C). These results collectively indicate that MCL-1i not only targets tumor cells directly but also reshapes the TME to support a more immunogenic tumor microenvironment.

**Fig. 6:**
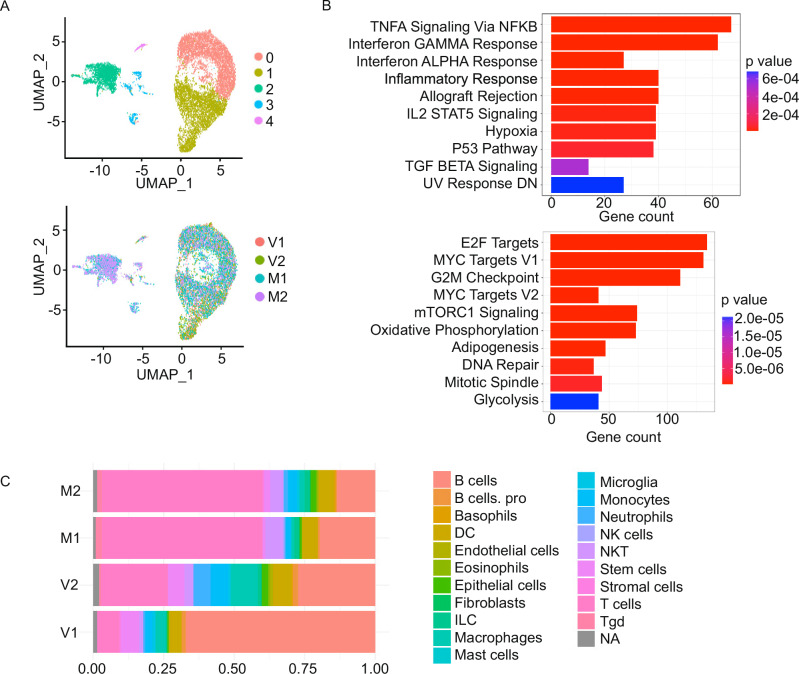
Single-Cell RNA-seq reveals tumor-intrinsic and microenvironmental remodeling following MCL-1 inhibition in MYC-associated B-cell lymphoma. **A** scRNA-seq analysis of control (V1 and V2) and MCL-1i-treated tumors (M1 and M2) from Eμ-MYC B cell lymphoma murine model and UMAP projection colored by graph-based cell clusters (upper panel) and experimental groups (lower panel). V: vehicle control, M: S63845 treatment. **B** Enrichment analysis by Enrichr for top ten enriched pathways of DEGs in MCL-1i-treated tumors (upper) and the control-treated tumors (lower). **C** The average percentages of tumor-infiltrating immune populations identified in A are shown for each group based on scRNA-seq analysis.

Next, we investigated whether intrinsic biological programs activated in cancer cells during MCL-1i therapy contribute to the modulation of the TME immune response in vivo. In parallel with the previously discussed immune profiling, we performed scRNA-seq on spleen lymphoma tissues from the Eμ-MYC B-cell lymphoma mouse model to assess MCL-1i-induced transcriptomic alterations in both tumor-intrinsic and TME-extrinsic compartments in an immunocompetent, syngeneic context. Comparative scRNA-seq analysis of vehicle control-treated (V1 and V2) and post-MCL-1i-treated (M1 and M2) Eμ-MYC lymphoma samples allows us to dissect the transcriptional dynamics and clonal evolution of lymphoma cells following treatment. Clustering of lymphoma cells revealed 5 distinct populations (Fig. 6A and Supplementary Fig. 6A). Graph-based clustering of the scRNA-seq data showed a redistribution of lymphoma cell populations post-MCL-1i treatment. Differential expression analysis identified significantly upregulated genes in residual lymphoma cells (cluster 2) after S63845 treatment, compared to controls. This included the enrichment of pathways related to interferon-alpha and interferon-gamma response, inflammation, and TNF-NF-ĸB signaling, along with the downregulation of MYC and upregulation of STAT1 (Fig. 6B and Supplementary Fig. 6B). In contrast, untreated lymphoma cells (cluster 0 and 1) exhibited significant enrichment in E2F, mTORC1 and MYC-related pathways, along with increased Ki-67 expression indicating active proliferation (Fig. 6B and Supplementary Fig. 6B). These in vivo findings are consistent with our earlier in vitro observations in parental and resistant lymphoma cell lines. In addition to tumor-intrinsic changes, we examined the evolution of non-lymphoma cell types within the TME. Using established marker genes, scRNA-seq clusters were annotated as immune cells, TME-associated myeloid cells, and lymphoma cells (Fig. 6C). MCL-1i significantly remodeled the TME, promoting increased infiltration of T cells and NK cells and reprogramming the cellular landscape towards a more immunogenic phenotype (Fig. 6C). Together, these results indicate that MCL-1i treatment not only exerts direct anti-tumor effects, debulking the tumor, but also reinvigorates the TME towards a more immune-permissive state in this syngeneic MYC-driven lymphoma model.

In addition, we evaluated the effect of MCL-1i on CAR-T cell viability and activity. As shown in Supplementary Fig. 6C, D, a sublethal dose of MCL-1i had minimal cytotoxic impact on CD19 CAR-T cell viability but enhanced CAR-T cell activities measured by flow cytometric CD69 surface expression and IFN-γ secretion. Together, these data indicate that targeting MCL-1 with low doses of MCL-1i therapy spares CAR-T cells while inducing immunogenic CAR-T activation and debulking the lymphoma tumor load, thus providing a therapeutic vulnerability to overcome CAR-T resistance.

### MCL-1 inhibition and CD19 CAR-T cell therapy reciprocally enhance efficacy and overcome resistance in aggressive B-cell lymphoma

**Fig. 7:**
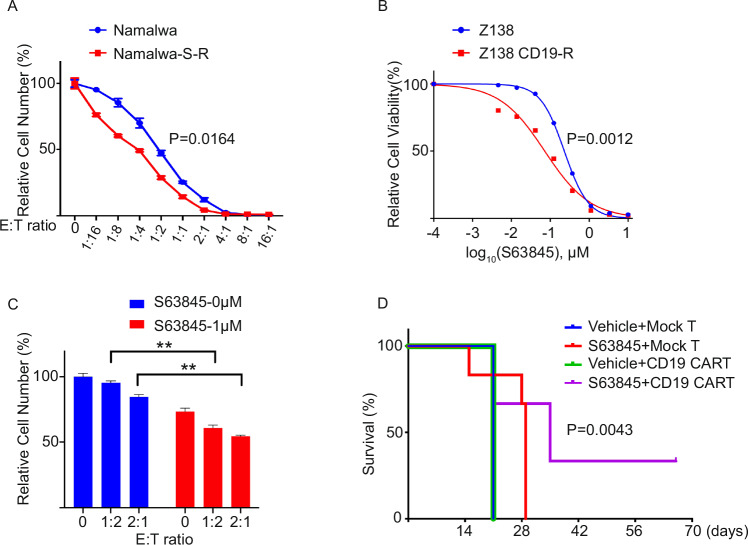
Combination of MCL-1 inhibition and CD19 CAR-T therapy overcomes resistance in aggressive B-cell lymphoma. **A** Cell cytotoxicity assay showing CD19 CAR-T cell-mediated killing in paired S63845-sensitive parental and S63845-resistant lymphoma cell lines. **B** Cell viability assay assessing the dose response of S63845 in paired CD19 CAR-T sensitive and resistant lymphoma cells. **C** Cell cytotoxicity assay shows the response of combination of S63845 and CD19 CAR-T in CD19 CAR-T resistant cells (CD19-R). **D** Survival analysis of mice treated with indicated therapies; statistical analysis was determined using the Mantel-Cox test. *n* = 6 for each group.

As shown above, lymphoma cells that develop drug resistance to the MCL-1i S63845 demonstrate upregulation of STAT1-mediated type I IFN and inflammatory signaling, leading to a reinvigorated and immunogenic TME. To explore whether these MCL-1i-resistant lymphoma cells remain susceptible to immunotherapy, we assessed the cytotoxic activity of CD19 CAR-T against MCL-1i-resistant cell lines. We generated CD19 CAR-T cells incorporating the CD28 costimulatory domain and evaluated their effect on MCL-1i-resistant lymphoma cells. As shown in Fig. 7A, CD19 CAR-T cells display potent cytotoxic activity against MCL-1i-resistant lymphoma lines. Given previous reports that CAR-T-resistant lymphomas exhibit MYC activation and immunosuppressive TME [42, 43], we next tested whether MCL-1i treatment could trigger apoptosis in CAR-T-resistant lymphoma cells. We generated CD19 CAR-T-resistant lymphoma lines by treating NanoLuc luciferase (Nluc)-tagged lymphoma cells with one or two rounds of CAR-T exposure for 4-6 weeks (Supplementary Fig. 7A). Notably, these CAR-T-resistant cells exhibited strong sensitivity to a sublethal dose of S63845, as shown in Fig. 7B. These findings demonstrate a reciprocal vulnerability: MCL-1i sensitizes tumors to CAR-T killing, while CAR-T-resistant cells respond to MCL-1 inhibition. To further investigate the potential synergy between MCL-1i and CAR-T cells in overcoming CAR-T resistance, we treated the CAR-T-resistant lymphoma cells with the combination of S63845 and CD19 CAR-T cells. Compared to either treatment alone, the combination treatment elicited significantly greater lymphoma cell killing, indicating a synergistic effect in CAR-T-resistant settings (Fig. 7C). To extend these findings in vivo, we employed an immunocompetent Eμ-MYC lymphoma mouse model, for which we generated and administered mouse CD19 CAR-T cells. Mice were randomized into 4 treatment groups: vehicle, S63845 alone, CD19 CAR-T cells alone, or the combination of S63845 followed by CD19 CAR-T cells. Mouse survival was monitored for up to 60 days (Supplementary Fig. 7B). MCL-1i enhanced both mock T and CD19 CAR-T cell infiltration into the lymphoma tumor bed, suggesting that S63845 does not impair T cell viability in vivo (Supplementary Fig. 7C). Kaplan-Meier survival analysis revealed that mice treated with combination therapy showed significantly prolonged survival compared to all other groups (Fig. 7D). These results suggest that a combination of MCL-1 inhibition and CD19 CAR-T therapy represents a promising and synergistic strategy for treating aggressive B-cell lymphomas.

## Discussion

This study identifies MCL-1 inhibition as a promising anti-tumor and immune-sensitization strategy, exerting its effects through both direct lymphoma cell killing and the activation of anti-tumor immunity. We established that combining MCL-1i with immunotherapeutic approaches represents a rational and effective strategy for the treatment of DLBCL and other aggressive B-cell lymphomas. These findings support the concept that MCL-1i enhances anti-tumor immune response and offers a novel approach to relieving tumor-induced immunosuppression. Moreover, this strategy has the potential to overcome CAR-T therapy resistance to improve the durability and efficacy of CAR-T therapy in MYC-driven lymphomas. Here, we have demonstrated that MCL-1i triggers STAT1-IFN inflammatory pathway activation, remodeling the TME and unleashing anti-tumor immunity in MYC-associated aggressive B-cell lymphomas. While MCL-1i treatment effectively eliminates the bulk of lymphoma cells, residual resistant lymphoma cells persist within a highly immunogenic TME, primed for T-cell adoptive immunotherapy. In addition, MCL-1i treatment and CD19 CAR-T reciprocally enhance their efficacy in a manner that may help to overcome monotherapeutic resistance. The combinatorial “one-two-punch” offers a promising path to better eliminate residual disease and lead to deep treatment responses in aggressive B-cell lymphomas.

Drugs targeting the MCL-1 protein are under preclinical and clinical development either as single agents or in combination with immunotherapy for patients with multiple cancers [44]. However, MCL-1i single-agent treatment is also invariably associated with the rapid acquisition of resistance. Here, we report MCL-1 as a major molecular determinant of cell survival and vulnerability in MYC-associated lymphomas and reveal MYC downregulation and STAT1 activation in both MCL-1i DTP and resistant cells. Furthermore, we have demonstrated that MYC activation and STAT1 knockdown attenuate lymphoma cell sensitivity to MCL-1i treatment, indicating that the MYC-STAT1 axis at least partially contributes to MCL-1i resistance evolution. Accordingly, MCL-1 is required to counteract MYC-driven sustained proliferation and associated apoptosis. Our data are consistent with a model in which, upon MCL-1 inhibition, lymphoma cells with high MYC lose this necessary anti-apoptotic survival signal and undergo apoptosis, while lymphoma cells with low MYC and less reliance on MCL-1 may survive and develop into MCL-1i DTP and resistant states. In line with our findings, loss of MYC signaling in DTPs from across various cancer types has been shown to induce a reversible, diapause-like DTP phenotype [45–47]. These observations support the possibility that the use of evolutionarily conserved embryonic survival strategies might underlie the ability of cancer cells to survive the hostile environment created by exposure to chemotherapy and/or targeted agents. Notably, as opposed to drug resistance, DTP populations manifest as a state in which a subset of tumor cells survives, but do not proliferate during active treatment [38, 48]. As a nexus of therapy resistance, this reality must be considered in designing more effective combination therapies.

In addition, we’ve shown that inhibition of MCL-1 potentiates the antitumor immune response by rewiring lymphoma cells to a more immunogenic state. Indeed, we observed that knockdown or pharmacological inhibition of MCL-1 activates type I IFN signaling, leading to increased transcription of pro-inflammatory cytokines and T-cell trafficking chemokines (CXCL9, CXCL10, CXCL11). Mechanistically, we demonstrated that MCL-1i-triggered MYC downregulation contributed to enhanced STAT1 activation, STAT1-mediated IFN tumor immunogenicity and T-cell infiltration via CXCL9/10/11 upregulation. In vivo, MCL-1i therapy led to reduced tumor growth with increased effector immune cell infiltration and increased inflammatory responses in MYC-associated B-cell lymphoma. These data are consistent with previous findings that MYC contributes to an immunosuppressive milieu of TME and that STAT1 activation can drive anti-tumor immunity in MYC-associated B-cell lymphomas [12, 49]. These findings also suggest that MCL-1i treatment induced DTP, and resistant cells are characterized by enhanced immunogenicity and are conducive to subsequent immunotherapy.

One of the key findings in our study is that MCL-1i treatment and CD19 CAR-T reciprocally overcome their acquired therapy resistance in aggressive B-cell lymphoma. First, this study demonstrated that drug-resistant lymphoma cells activate STAT1-Type I IFN and inflammatory signaling pathways following MCL-1i treatment, and that the TME was reinvigorated towards an immunogenic, anti-tumor immunity in MYC lymphoma in vivo. In this manner, MCL-1i treatment primed lymphoma cells for response to CAR-T therapy. CD19 CAR-T therapy was also demonstrated to be highly effective against MCL-1i-treated and resistant lymphoma cells. Second, we and others have shown that potential mechanisms hampering CAR-T therapy efficacy include intrinsic genetic and epigenetic dysregulation in lymphoma cells, especially MYC activation and the extrinsic immunosuppressive TME, such as enriched myeloid cells and reduced T-cell infiltrates [35, 36]. Specifically, dysregulation of apoptosis and the immunosuppressive TME are primary mechanisms of CAR-T therapy success. Consistent with those prior observations, we’ve demonstrated here that MCL-1i can reverse CAR-T resistant phenotypes by remodeling the TME toward an immunogenic milieu, enhancing T-cell trafficking and reducing the number of immunosuppressive myeloid cells, thus overcoming CAR-T resistance. Together, these findings provide a strong rationale for using MCL-1 targeting to modulate apoptosis and remodel the TME to overcome CAR-T resistance and enhance CAR-T therapy efficacy in aggressive lymphomas.

Finally, we propose that further development and optimization of combinatorial therapeutic strategies involving MCL-1i followed by immune-CAR-T therapy represents a rational approach to prolonging remissions and potentially achieving a cure of aggressive MYC-associated lymphomas. The combinatorial strategy of MCL-1i followed by CAR-T is particularly well-suited for aggressive lymphomas, given that the tumor load and myeloid-related immunosuppressive TME are major obstacles for CAR-T therapy. In this context, MCL-1i treatment initially serves to reduce the bulk of the lymphoma system tumor burden, while residual DTP and resistant lymphoma cells and the surrounding TME become immunologically primed for subsequent CD19 CAR-T therapy, which can then achieve more complete tumor cell clearance. In line with the above proposal, Lee et al [50]. demonstrated that a combination of CD19 CAR-T with the FDA-approved BCL-2 inhibitor venetoclax resulted in in vivo synergy in venetoclax-sensitive lymphomas and that bridging therapy with venetoclax improves the efficacy of CD19 CAR-T immunotherapy in patients with MCL [51]. Most combinations of current chemotherapy with targeted agents remain largely empirical and are susceptible to acquired therapy-induced resistance upon continuous treatment. In contrast, rational combination therapies such as that proposed here hold the potential for preventing or delaying therapy-induced resistance during the initial tumor response by anticipating and then counteracting the inherent plasticity of aggressive lymphoid cancers. The crux of this strategy is the ability of BH3 mimetics, such as BCL-2 and MCL-1 inhibitors to engage the immune system and act beyond tumor cells in a manner that will hopefully provide lasting benefits to cancer patients via enhanced and long-term immunosurveillance of minimum residual disease.

## Supplementary information

Supplementary Information

## Data Availability

The scRNA-seq data generated in this study have been deposited in the NCBI Gene Expression Omnibus (GEO) under accession number GSE314230. Bulk RNA-sequencing data have been deposited under accession number GSE314603.
