# Supplementary material for: Synthetic lethality of MCL-1 inhibition and CAR-T therapy in aggressive B-cell lymphoma
Source: Leukemia. 2026 Feb 12;40(3):638–48. doi: 10.1038/s41375-026-02884-8 (PMC12960227; doi:10.1038/s41375-026-02884-8)
Supplement: Supplementary file 1 — Supplementary Information [file 41375_2026_2884_MOESM1_ESM.pdf]

Supplementary Information

Synthetic Lethality of MCL-1 Inhibition and CAR-T Therapy in Aggressive B-cell Lymphoma

Jing Gao, Xiaohong Zhao, Qing Yin, Allen Hu, Kevin Qiu, Loryn Blackburn, Lenny Lei, Rui Xiong, Chengfeng Bi, Jeffrey W. Craig, Craig A. Portell, Marco L. Davila, Michael E. Williams, Jianguo Tao.

Supplemental Methods

Small Molecule Inhibitors

S63845 (Selleckchem, Cat# S8383) agents was dissolved in DMSO to a final concentration of 10 mM, aliquoted, and then stored at -20°C.

Patients and Tumor Specimens

Fresh primary lymphoma samples from MCL patients were obtained from peripheral blood following informed consent and with approval by the University of Virginia Institutional Review Board. Lymphoma cells were isolated by Ficoll-Plaque purification, and only samples consisting of greater than 80% tumor cells were used for experiments.

Generation of S63845 Resistant Cell Lines

To establish the S63845 resistant cell lines, MCL (HBL-2 and Mino), DLBCL (DHL-16) and BL (Namalwa) cells were grown in RPMI-1640 medium with 10% FBS and treated with vehicle control or escalating dose of S63845 for approximately 3 months. Before

exposing cells to S63845, cells were maintained in suspension. Trypan blue staining and cell viability assay in suspension cells were used to determine viability every 2-3 weeks and S63845 concentrations were increased if the viability of cells was >65%. If cell viability was <65%, populations were expanded in suspension in drug-free medium and then re-exposed to the same concentration of S63845. After ~90 days, drug-resistant variants emerged, and IC50s were determined every two weeks. Following another 30 days of selection, stable variants emerged referred to as S-R. Drug-resistant variants were maintained in drug free medium for 3 days before being used in experiments. Cells exposed to DMSO were maintained in parallel and used for comparison with their respective drug-resistant cell lines. We used at least 10 × IC50 (ranging from 10 to 100-fold) as the threshold to define resistance.

Cell Viability Assays

5000 cells were seeded in 96-well plates in 50µl medium. 50µl drug medium at nine serial diluted concentrations was added to the cell suspension in each well. Each condition on these plates was seeded in triplicate. After a 72-hour incubation, 20ul of Resazurin reagent (R&D Systems, Cat# AR002) was added into each well. After a 2-hour incubation at 37°C in 5% CO_2, the plates were read using 544/590 nm wavelengths to estimate cell proliferation. Relative cell viability was normalized to DMSO treated wells.

MYC and STAT1 knockdown

pLentiCRISPR v2 plasmids containing sgRNA sequences, along with lentiviral packaging vectors PsPax2 and envelope plasmid pMD2.g, were transfected into HEK-293T cells

using Lipofectamine 2000 (Invitrogen). Lentiviral supernatants were collected and filtered. 48 hours and 72 hours after transfection, culture supernatants containing virus were harvested and filtered. Cells were infected with the concentrated viral supernatants and 8μg/mL polybrene. To make a stable knockdown, 3 days after viral infection, cells were selected in 0.5μg/mL puromycin. Puromycin resistant cells were picked up and characterized by western blot to determine if MYC and STAT1 was significantly reduced after transduction and selection. gRNA sequence for MYC is GCATCGTCGCGGGAGGCTGC and STAT1 is GCTGAATTTCGGCACCTGGT.

RNA isolation and quantitative real time polymerase chain reaction (qRT-PCR)

Total RNA was isolated using RNA isolation kit, RNeasy Plus Mini (Qiagen, Cat# 74134), cDNA was synthesized using the Verso cDNA Synthesis kit (Thermo Scientific, Cat# A1453B) according to instructions of the manufacturer. Quantitative real-time PCR (qRT-PCR) was performed using SYBR™ Green PCR Master Mix (Applied Biosystems, Cat# A46109) for indicated target genes and GAPDH was used as an internal sample probe to control for inter-assay variability. Relative quantification of expression levels was performed according to the comparative threshold cycle (Ct) method assuming equal efficiency of target and housekeeping genes. Primer sequences: GAPDH (Forward: 5'-ACCCAGAAGACTGTGGATGG-3'; Reverse: 5'-TTCAGCTCAGGGATGACCTT-3'), STAT1(Forward: 5'-CCGTTTTCATGACCTCCTGT-3'; Reverse: 5'-TGAATATTCCCCGACTGAGC-3'), CXCL9 (Forward: 5'- TTTTCCTCTTGGGCATCATC-3'; Reverse: 5'-GAACAGCGACCCTTTCTCAC -3'), CXCL10 (Forward: 5'-GTGGCATTCAAGGAGTACCTC-3'; Reverse: 5'-GCCTTCGATTCTGGATTCAGACA-3'),

CXCL11 (Forward: 5’- AGAGGACGCTGTCTTTGCAT-3’; Reverse: 5’-TGGGATTTAGGCATCGTTGT-3’), IFNA1(Forward: 5’-CTCATACACCAGGTCACGCT-3’; Reverse: 5’- AGTGTAAAGGTGCACATGACG-3’), IFNAR1 (Forward: 5’-CGCCTGTGATCCAGGATTATCC-3’; Reverse: 5’-TGGTGTGTGCTCTGGCTTTCAC-3’), IFNB1 (Forward: 5’-CATTACCTGAAGGCCAAGGA-3’; Reverse: 5’-CAATTGTCCAGTCCCAGAGG-3’), IFNE1 (Forward: 5’-GTCTTTGAGTCCTCAGCAGTACC-3’; Reverse: 5’- TCCGTGTGGTTTTCCTCCCAAC-3’), IFNW1 (Forward: 5’-CAGGAGATGGTAAAAGGGAGCC-3’; Reverse: 5’-GAAGTCCAGTGTGGAGTTGGTC-3’), IRF-1 (Forward: 5’-GCCATTCACACAGGCCGATA-3’; Reverse: 5’- GTGGAAGCATCCGGTACACT-3’), IRF-2 (Forward: 5’-AAGCACACTGAGAGGGCAC-3’; Reverse: 5’-CATCCCACCCATGTCTAGCC-3’), IRF-5 (Forward: 5’-TAGAGGCTACCCAGGAGCAA-3’; Reverse: 5’- GCCCACTCCAGAACACCTTA-3’), IRF-7 (Forward: 5’-TGTGCTGGCGAGAAGGC-3’; Reverse: 5’- TGGAGTCCAGCATGTGTGTG-3’), IRF-9 (Forward: 5’-TTCTTCAAGGCCTGGGCAAT-3’; Reverse: 5’-CCTGGTGGCAGCAACTGATA-3’), and TYK2 (Forward: 5’-GGTTGACCAGAAGGAGATCACC-3’; Reverse: 5’- TCCTCGTCATCCATCTTGCCCT-3’).

Chromatin immunoprecipitation (ChIP)

Chromatin immunoprecipitation (ChIP) assaying was performed using a ChIP-IT Express Kit (Active Motif, Cat# 53008) according to the manufacturer's protocol. Cells were fixed with 1% formaldehyde/PBS for 10 min. DNA was sonicated to 500-1,000 bp for all experiments. Immunoprecipitated DNA enrichment was normalized to the input. The

antibodies used were STAT1 antibody (Cell Signaling Technology, Cat# 9172). Normal rabbit IgG Cell Signaling Technology, Cat# 2729) was used as a negative control for each assay. The primer set for qPCR was as follows: CXCL9 (Forward: 5'-GGAACCACACAGGGAAGGAG-3'; Reverse: 5'- GCCTTCTGGGGTTTTTGTTCC-3'), CXCL10 (Forward: 5'- ACTACGTATTTGCAATTGAATGAACT-3'; Reverse: 5'-GGTGCCCAGCCAAGAATT-3'), CXCL11 (Forward: 5'-TGGAGGTTGAAGAATCCACAGG-3'; Reverse: 5'- TCCTGTAGCATGACTCTGGC-3'), IFNA1 (Forward: 5'- ACAATCTCGGCTCACTGCAA-3'; Reverse: 5'-CTAGGCATGGTTGTGTGCAC-3'), IFNB1 (Forward: 5'- TAGGAACTATGTGGCGTCCG-3'; Reverse: 5'- CTAAGCCTGCTTCCAGTCCC-3'), IFNE1 (Forward: 5'-TGTCTCTAGCAATTTCTGGCA-3';Reverse: 5'-TGGGTAGATACAATAAAATATGCCACT-3'), IFNW1 (Forward: 5'-TCTTTGTAGTGTTTCATATAAGCGAA-3'; Reverse: 5'-GGTGAATAAACACAGTTTCTTTCCG-3'), IRF-1 (Forward: 5'-GCCAGACACTGAAGAGGGAG-3'; Reverse: 5'- TGACATATGACAATGCCCCCT-3'), IRF-5 (Forward: 5'- TTCAGAACCACAGGTCGACG-3'; Reverse: 5'-TCATCCTCAAACCCTGCACC-3'), IRF-7 (Forward: 5'-ACTTGGAAGATAAAGCACTTGGTG-3'; Reverse: 5'- AGGTACACAGCAGCTGTTGG-3'),IRF-9 (Forward: 5'- ACTGTGGGGGAATGCAGATG-3'; Reverse: 5'-CCCTGCCTCCCTCAGATACT-3'), TYK2(Forward: 5'- ACAGACACCACCTCATGAGC-3'; Reverse: 5'- TGTGACCTCAGGCTAATGGC-3').

Western Blot

Cells were lysed in RIPA Lysis and Extraction Buffer (Thermo Scientific, Cat# 89901) with a Protease and Phosphatase Inhibitor Cocktail (Thermo Scientific, Cat# 78446) for 30 minutes on ice. The BCA protein assay kit (Thermo Scientific, Cat# 23227) was used to determine protein concentrations. 30 µg of protein lysates were separated by sodium dodecyl sulfate polyacrylamide gel electrophoresis (SDS-PAGE) and transferred to PVDF membrane (Bio-Rad Laboratories, Cal# 1620177). After blocking with 5% non-fat milk in 1 × TBST, membranes were incubated with primary antibodies overnight at 4°C, followed by secondary antibodies conjugated with horseradish peroxidase (HRP) for 1 hour at room temperature. Protein bands visualized using Pierce™ ECL Western Blotting Substrate (Thermo Scientific, Cat# 32106) and imaged with an iBright imaging system. The antibodies used in western blot were as follows: MYC (Abcam, Cat# ab32072, 1:10000), β-actin (Santa Cruz Biotechnology Inc., Cat# sc-47778HRP, 1:2000), p-STAT1 (Ser727) (Cell Signaling Technology, Cat# 9177, 1:1000), STAT1 (Cell Signaling Technology, catalog 9172, 1:000), MCL-1 (Cell Signaling Technology, Cat# 94296, 1:1000) and cleaved PARP (Cell Signaling Technology, Cat# 5625, 1:1000).

Luminescence-Based Cytotoxicity Assay

Target cells were transduced with a luciferase reporter and seeded in 96-well plates at 1 × 10^4 cells/well. Effector cells (CD19 CAR-T cells) were added at various effector-to-target (E:T) ratios in triplicates. After co-culture for 48 hours at 37 °C, cell lysis and luciferase activity were measured using the ONE-Glo™ Luciferase Assay System (Promega, Cat# E6110) following the manufacturer's instructions. Luminescence was read on a microplate reader (SpectraMax iD3).

Flow cytometry-based cytotoxicity assay

Target cells are fluorescently labeled with eFluor 450 dye (Invitrogen, Cat# 65-0842-85) and seeded to 96-well culture plates. CD19 CAR-T cells were added to target cells at the designated E:T ratios and gently mixed. After co-culture for 48 hours at 37 °C, a forward/side scatter gate was drawn to capture the lymphocyte population followed by an enumeration of live CD19 CAR-T (7AAD−/eFluor 450−) and live target cells (7AAD−/eFluor 450+). The percent killing was calculated using the following formula:

$$\left[1 - \frac{\text{number of targets cultured with effectors}}{\text{number of targets cultured without effectors}}\right] \text{x } 100$$

Generation of CD19 CAR-T Cells

Peripheral blood mononuclear cells (PBMCs) were isolated from healthy human donors using Ficoll-Paque density gradient centrifugation. $CD3^+$ T cells were enriched by negative selection using the EasySep™ Human T Cell Isolation kit (STEMCELL Technologies, Cat# 17951) and subsequently activated with anti-CD3/CD28 Dynabeads (Thermo Scientific, Cat# 11131D) in the presence of human IL-2 (STEMCELL Technologies, Cat# 78036.1, 90IU/ml) for 48hours prior to transduction. Activated T cells were transduced with CD19-directed CAR lentivirus using RetroNectin (Takara, Cat# T100B) -coated non-tissue culture-treated plates by spinoculation at 2000 × g for 60 minutes at room temperature, repeated on two consecutive days. Following transduction, cells were maintained in fresh complete RPMI-1640 medium supplemented with 10% FBS and IL-2 (90IU/ml), and expanded for 10–12 days, keeping the cell density between 0.5 and 1 × 10^6cells/ml.

Mouse T cells were isolated from spleens of naïve mice by mechanical dissociation through a 40um cell strainer. T cells were enriched using the EasySep™ Mouse T Cell isolation kit (STEMCELL Technologies, Cat# 19851) and subsequently activated with CD3/CD28 Dynabeads (Thermo Scientific, Cat# 11453D) in the presence of IL-2 for 24 hours. Activated T cells were transduced by centrifugation on RetroNectin-coated plates at 2000 x g for 60 minutes, repeated on two consecutive days. Cells were then cultured in complete RPMI-1640 medium supplemented with 10% FBS and IL-2 for expansion.

Generation of CD19 CAR-T Resistant Lymphoma Cells

To generate CD19 CAR-T cell-resistant lymphoma cells, parental Z138 lymphoma cells were repeatedly co-cultured with donor-derived CD19 CAR-T cells at sublethal effector-to-target (E:T) ratios. Briefly, lymphoma cells were seeded in complete RPMI-1640 medium and co-cultured with freshly expanded CD19 CAR-T cells at an E:T ratio of 1:1 or 1:2. After 48–72 hours, surviving tumor cells were harvested, washed to remove remaining T cells, and expanded in fresh medium. This process was repeated for 3-5 cycles, with increasing CAR-T pressure to select for resistant populations. After the final round of selection, surviving tumor cells were maintained in culture for an additional 1-2 weeks in the absence of CAR-T cells to stabilize the resistant phenotype. These CAR-T-resistant cells were termed CD19-R (resistant to CAR-T). Phenotypic and functional resistance was validated by repeat cytotoxicity assays.

In Vivo Treatment of Eμ-MYC Lymphoma with CD19 CAR-T Cells and S63845

All experiments were approved by the University of Virginia Institutional Animal Care and

Use Committee. Eμ-MYC transgenic mice (C57BL/6 background; The Jackson Laboratory) were used to generate a syngeneic, immunocompetent B-cell lymphoma model. Single-cell suspensions of spontaneous lymphomas were harvested from lymph nodes of diseased Eμ-MYC mice, filtered through a 70 μm cell strainer, and resuspended in sterile PBS. A total of 1×10^6 viable lymphoma cells were injected intravenously (I.V.) via the tail vein into 6- to 8-week-old C57BL/6 recipient mice (Jackson Laboratory) on day 0. Mice were randomly assigned to one of four treatment groups (n =8 per group) on day 6 post-tumor inoculation: (1) Vehicle + Mock T cells, (2) S63845 + Mock T cells, (3) Vehicle + CD19 CAR-T cells, and (4) S63845 + CD19 CAR-T cells. CD19 CAR-T cells and corresponding mock T cells were generated from C57BL/6 mouse splenocytes as previously described. The MCL-1 inhibitor S63845 was first dissolved in 100% DMSO to prepare a 50 mg/mL stock solution and then diluted with 2% TPSG vehicle (Tween-80:PEG300:0.9% NaCl, 1:1:8 v/v) to the final injection concentration. The final working solution contained <10% DMSO and was freshly prepared before each use. S63845 was administered by tail vein injection (i.v.) at a dose of 25 mg/kg once per week on days 7, 14, and 21. Control groups received vehicle (10% DMSO, 40% PEG300, 5% Tween-80 in saline) on the same schedule. CD19 CAR-T or mock T cells (5×10^6 cells per mouse) were injected intravenously on day 10. Mice were monitored daily for signs of disease progression and survival. In some experiments, spleens were harvested at defined time points post-treatment for flow cytometric analysis.

Flow Cytometry

Single-cell suspensions were prepared from mouse spleens by mechanical dissociation

followed by filtration through a 40 μm cell strainer. Red blood cells were lysed using ACK lysis buffer (Gibco, Cat# A1049201). Cells were washed and resuspended in flow cytometry staining buffer (PBS with 2% FBS and 2 mM EDTA). For surface marker staining, cells were first blocked with anti-mouse CD16/CD32 Fc block (BioLegend, Cat# 101320) for 10 minutes at 4 °C to prevent nonspecific binding, then stained with fluorophore-conjugated monoclonal antibodies for 30 minutes at 4 °C in the dark. The following antibodies were used: B220 (RA3-6B2, BioLegend, Cat # 103205, 1:200), CD45 (30-F11, BioLegend, Cat # 103112, 1:200), CD3 (145-2C11, BioLegend, Cat # 100308, 1:200), CD4 (RM4-5, BioLegend, Cat # 100515, 1:200), CD8 (53-6.7, BioLegend, Cat # 100737, 1:200), PD-1 (29F.1A12, BioLegend, Cat # 135208, 1:200), TIM-3 (RMT3-23, BioLegend, Cat # 119718, 1:200), CD25 (PC61, BioLegend, Cat # 102010, 1:200), GR1 (RB6-8C5, Biolegend, Cat # 108407, 1:200) and CD11b (M1/70, BioLegend, Cat # 101205, 1:200). Dead cells were excluded using a fixable viability dye (Zombie Aqua™, BioLegend, Cat # 423102). After staining, cells were washed and resuspended in staining buffer for analysis. Data were acquired using a BD LSRFortessa or Cytek Aurora flow cytometer and analyzed with FlowJo software (TreeStar, v10.8). Compensation was performed using single-stained controls, and gating was based on fluorescence minus one (FMO) control where necessary.

RNA-Sequencing

All samples were prepared in biological triplicates. Total RNA was extracted from samples using RNA isolation kit, RNeasy Plus Mini (Qiagen, Cat# 74134). After quality control, high-quality (Agilent Bioanalyzer RIN >8.0) total RNA was used to generate the RNA-seq

library. Sequencing libraries were prepared using TruSeq Stranded mRNA Library Prep Kit (Illumina Cat # 20020594) according to the manufacturer's instructions. Libraries were sequenced on Illumina NovaSeq X following Illumina's standard protocol using the Illumina cBot and HiSeq 3000/4000 PE Cluster Kit. Base calling was performed using Illumina's RTA software (version 2.5.2). Tophat2 was used to align the Fastq files. Differential expression analysis was performed using DESeq2 in R. Raw count data were directly used as the input. Genes with low expression across all samples were filtered out prior to analysis. DESeq2 was used to normalize the data, estimate size factors and dispersions, and perform differential testing using a negative binomial generalized linear model. Differentially expressed genes (DEGs) were defined as those with an adjusted *p*-value (Benjamini–Hochberg correction) < 0.05 and an absolute log2 fold change ≥ 1.5, unless otherwise stated. Visualization of DEGs was conducted using ggplot2 packages in R. Gene set enrichment analysis (GSEA) was performed using the clusterProfiler packages. For GSEA, all genes were ranked based on the DESeq2-derived statistic and pre-ranked GSEA was conducted against curated gene sets from the HALLMARK gene sets. Enrichment scores and normalized enrichment scores (NES) were calculated, and pathways with a false discovery rate (FDR) < 0.25 were considered significantly enriched, following GSEA standard guidelines.

Single-cell RNA-seq

Single-cell suspensions were prepared from Namalwa cells, and mouse spleen cells followed by filtration through a 40 μm cell strainer. Cell viability was assessed using trypan blue exclusion, and only samples with >85% viability was used for single-cell library

preparation. Single-cell RNA-seq libraries were generated using the 10x Genomics Chromium Single Cell 3' v3.1 Kit following the manufacturer's instruction. Raw sequencing data were processed using the Cell Ranger software. Downstream analysis was conducted in Seurat (version4.1.1) within R. Low-quality cells were filtered out based on the following thresholds: cells with fewer than 200 detected genes, more than 6,000 detected genes, or more than 10% mitochondrial gene content were excluded. Gene expression data were normalized using the NormalizeData function and scaled using ScaleData. Highly variable genes were identified with FindVariableFeatures, and data integration across samples was performed using Harmony. Dimensionality reduction was performed using principal component analysis (PCA) followed by Uniform Manifold Approximation and Projection (UMAP) for visualization. Cell clustering was conducted using the FindClusters function based on shared nearest neighbor (SNN) modularity optimization. Cell-type annotation was performed based on canonical marker genes and differential gene expression analysis using the FindMarkers function with the Wilcoxon rank-sum test. For pathway enrichment analysis, differentially expressed genes were analyzed using clusterProfiler for Hallmark pathway enrichment. In some cases, AUCell was used to score gene set activity at the single-cell level.

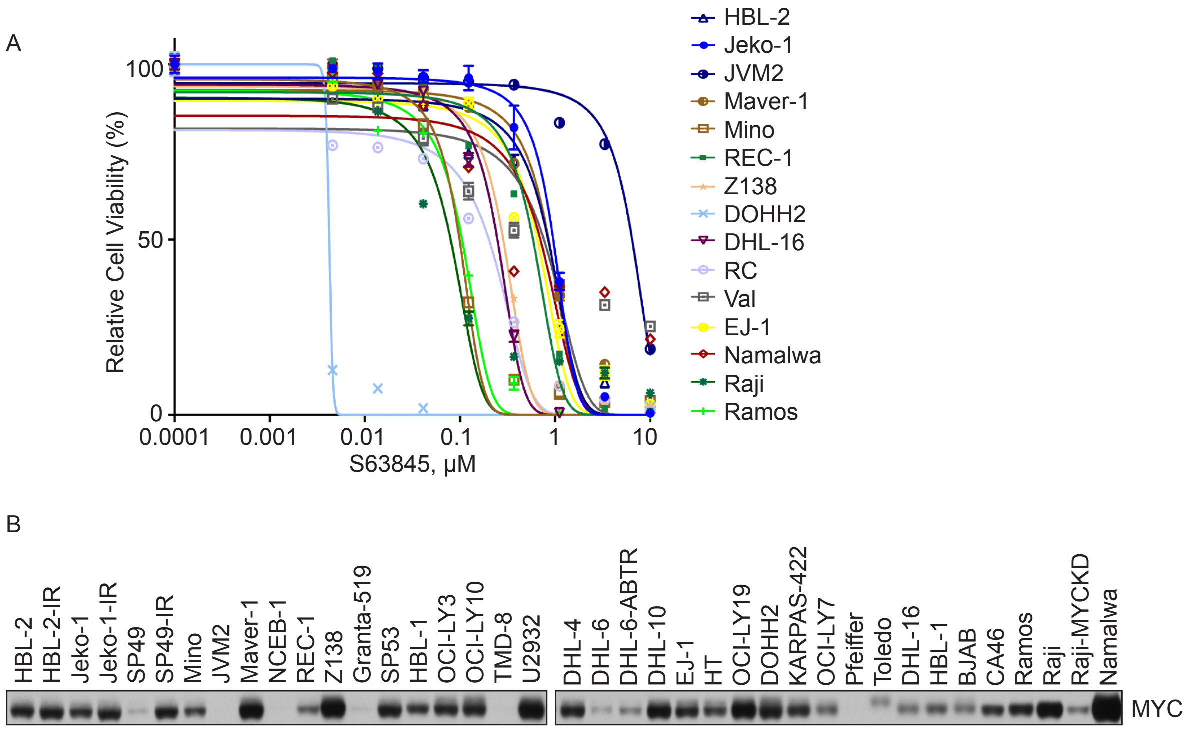

Supplementary Figure 1. Aggressive B-cell lymphoma cell lines are sensitive to MCL1 inhibition. A. Dose-response curves of MCL cell lines treated with S63845 for 72 hours. Data are presented as mean ± SD, from three technical replicates per cell line. **B.** Western blot analysis of MYC protein levels in B-cell lymphoma cell lines, β-actin protein abundance by western blot was previously published in our earlier work [37].

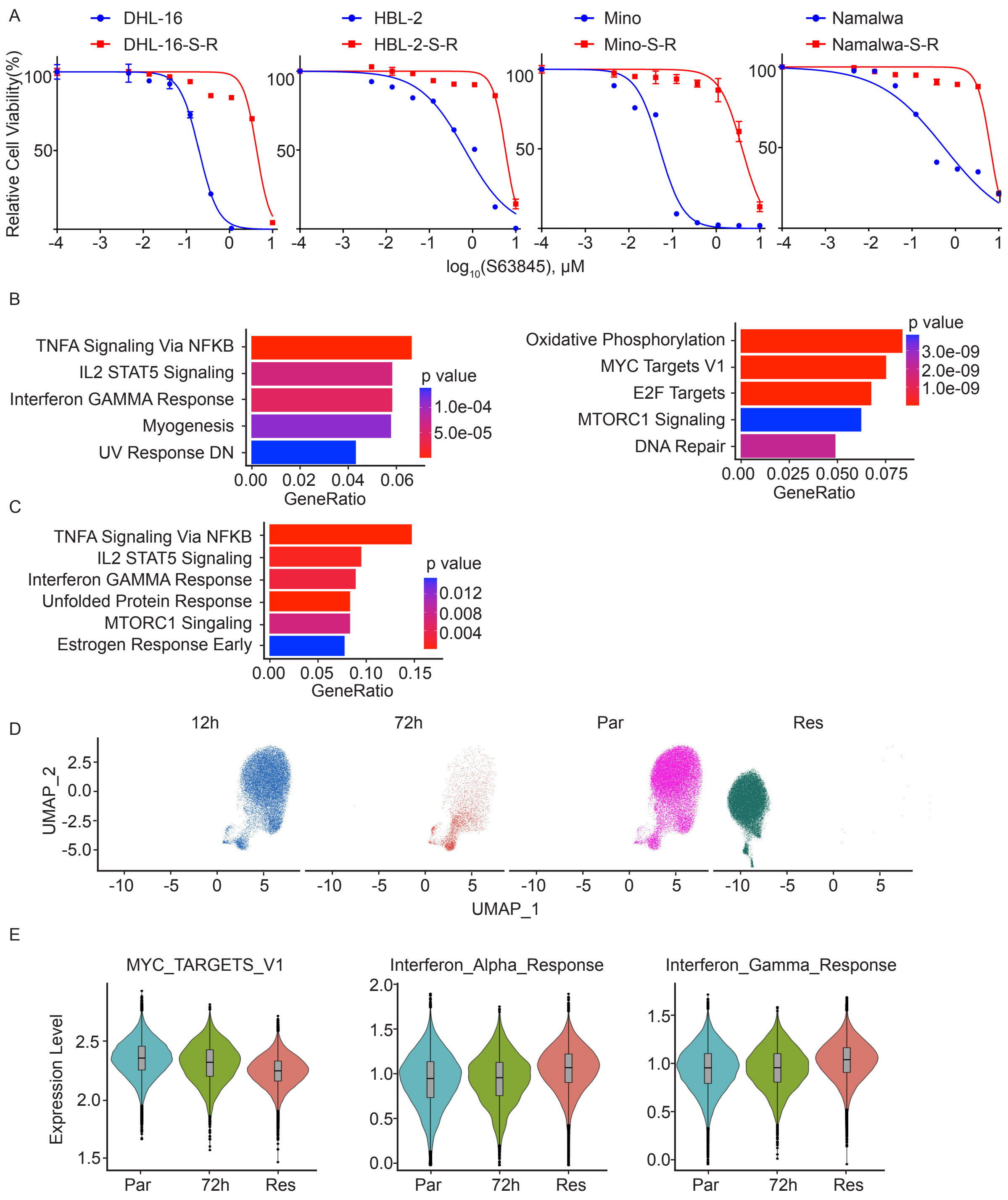

Supplementary Figure 2. Alterations in MYC and STAT1 signaling pathways during the development of MCL1i resistance. A. Cell viability assay showing the dose response curves of S63845 in paired sensitive parental and resistant lymphoma cell lines

(S-R). **B.** Primary patient sample treated overnight with S63845 compared to DMSO, showing representative positively (left) and negatively (right) enriched HALLMARK pathways. **C.** Common HALLMARK pathway alterations in HBL-2 and Mino cell liens treated with S63845 (IC_{50}) for 72 hours compared to untreated parental cells. **D.** scRNA-seq analysis of UMAP plot colored by graphed-base cell clusters in untreated (Par), 12 hours (12h), 72 hours (72h) and chronically MCL-1i treated (Res) Namalwa cells, respectively. **E.** Violin plots showing the AUCell enrichment of MYC Targets V1 decreased, Interferon-alpha and Interferon-gamma signaling pathways increased in 72 hours and chronically MCL-1i treatment in Namalwa cells. Data in panel **A** is representative of at least three independent experiments.

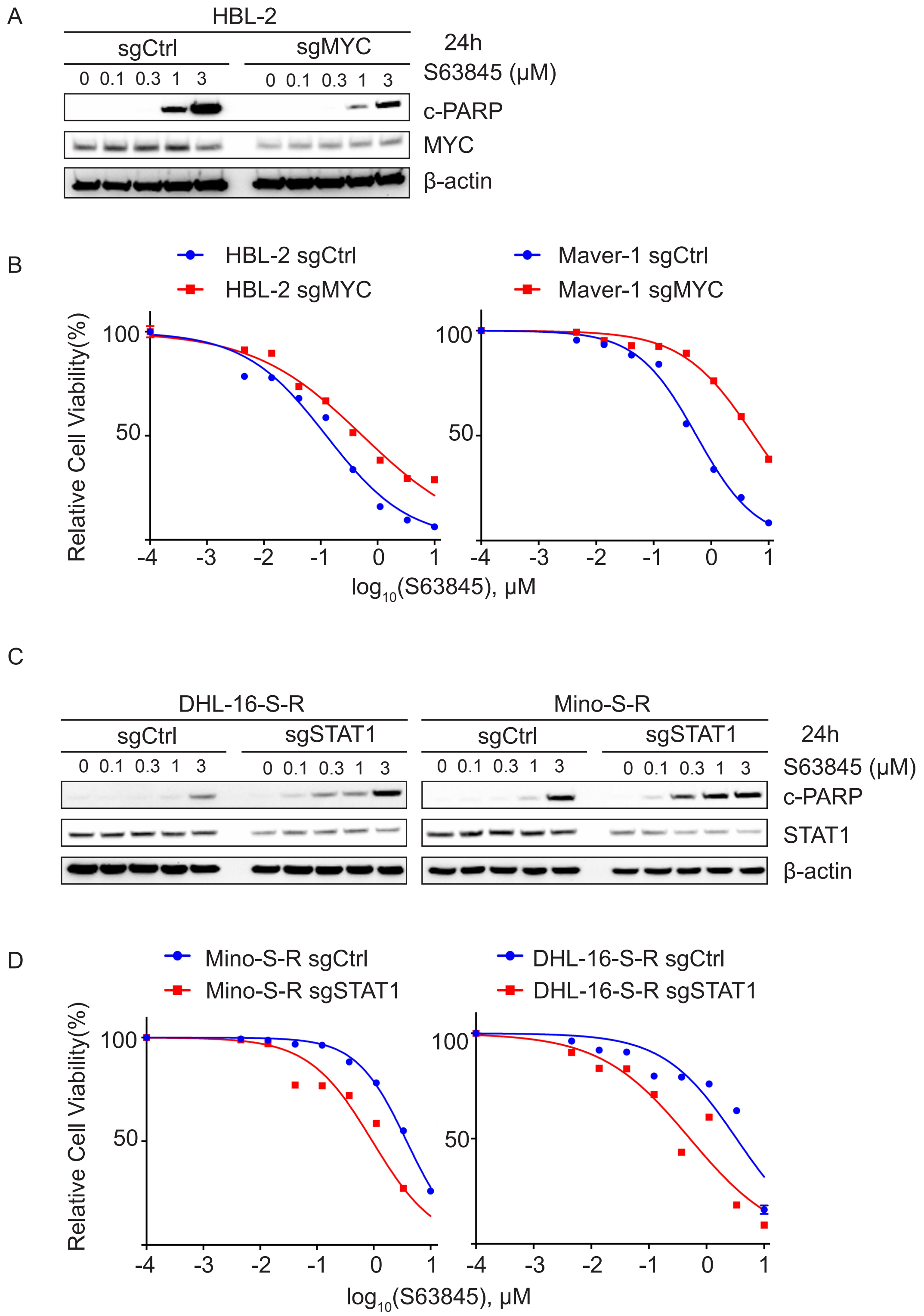
A
HBL-2
sgCtrl
sgMYC
24h
0 0.1 0.3 1 3
0 0.1 0.3 1 3
S63845 (μM)
c-PARP
MYC
β-actin
B
HBL-2 sgCtrl
HBL-2 sgMYC
Maver-1 sgCtrl
Maver-1 sgMYC
Relative Cell Viability(%)
100
50
-4 -3 -2 -1 0 1
log10(S63845), μM
C
DHL-16-S-R
Mino-S-R
sgCtrl
sgSTAT1
24h
S63845 (μM)
c-PARP
STAT1
β-actin
D
Mino-S-R sgCtrl
Mino-S-R sgSTAT1
DHL-16-S-R sgCtrl
DHL-16-S-R sgSTAT1
Relative Cell Viability(%)
100
50
-4 -3 -2 -1 0 1
log10(S63845), μM

Supplementary Figure 3. MCL-1i-induced MYC downregulation and STAT1 upregulation contribute to the development of resistance to MCL-1 inhibition. A. Western blot analysis of cleaved PARP (c-PARP) and MYC protein expression in parental (sgCtrl) and MYC knockdown (sgMYC) cell lines treated with the indicated concentrations of S63845 for 24 hours. **B.** Cell viability assay demonstrating enhanced proliferation of MYC knockdown cells (HBL-2 and Maver-1) following S63845 treatment compared to parental cells. **C.** Western blot analysis of cleaved PARP (c-PARP) and STAT1 expression in S63845-resistant cell lines (sgCtrl) and corresponding STAT1 knockdown cell lines (sgSTAT1) treated with the indicated doses of S63845 for 24 hours. **D.** Cell viability assay showing reduced proliferation in STAT1 knockdown S63845-resistant cell lines (DHL16 and Mino). Data shown in panels **B** and **D** are representative of at least three independent experiments.

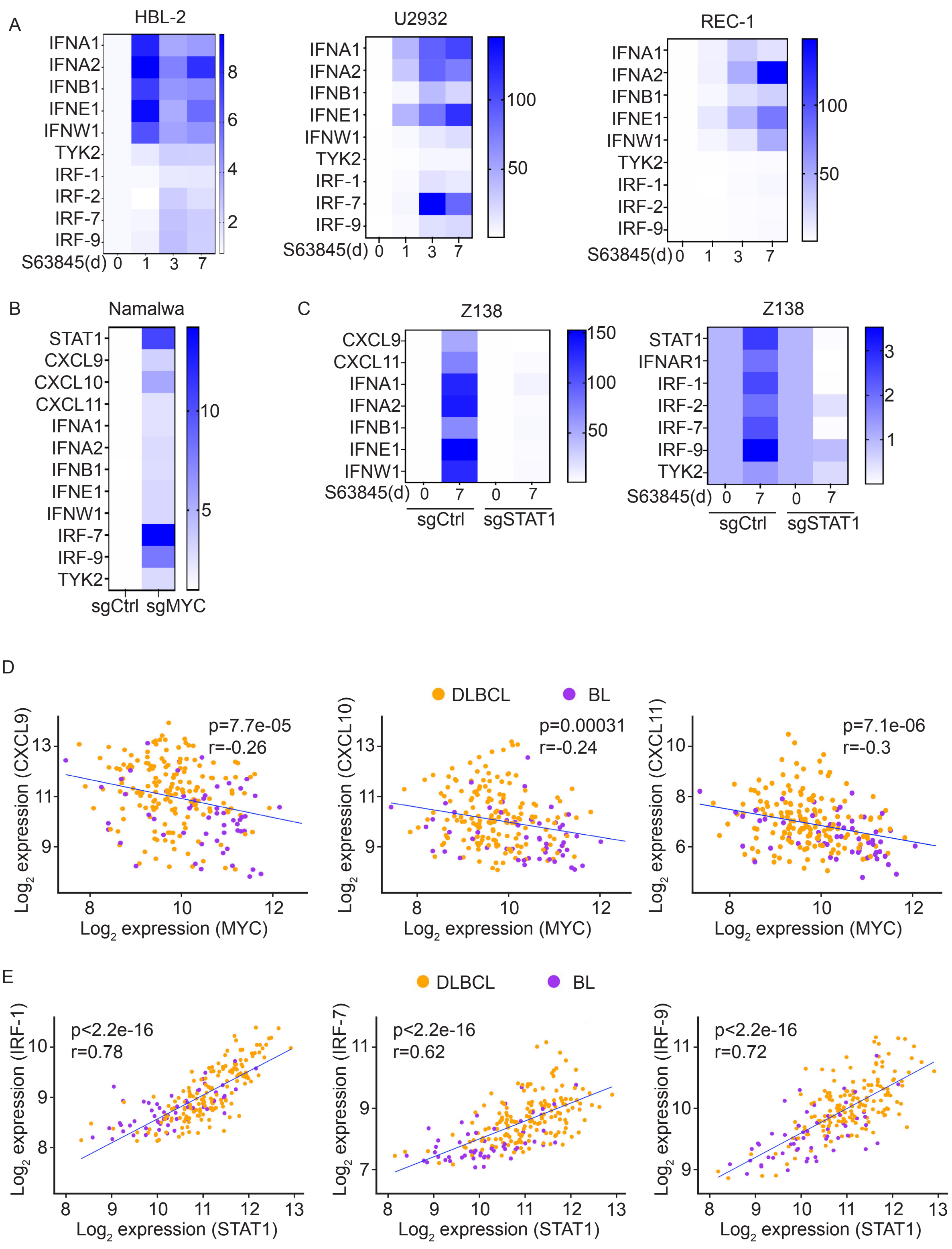
A
HBL-2
IFNA1
IFNA2
IFNB1
IFNE1
IFNW1
TYK2
IRF-1
IRF-2
IRF-7
IRF-9
S63845(d) 0 1 3 7
8
6
4
2
U2932
IFNA1
IFNA2
IFNB1
IFNE1
IFNW1
TYK2
IRF-1
IRF-7
IRF-9
S63845(d) 0 1 3 7
100
50
REC-1
IFNA1
IFNA2
IFNB1
IFNE1
IFNW1
TYK2
IRF-1
IRF-2
IRF-9
S63845(d) 0 1 3 7
100
50
B
Namalwa
STAT1
CXCL9
CXCL10
CXCL11
IFNA1
IFNA2
IFNB1
IFNE1
IFNW1
IRF-7
IRF-9
TYK2
sgCtrl sgMYC
10
5
C
Z138
CXCL9
CXCL11
IFNA1
IFNA2
IFNB1
IFNE1
IFNW1
S63845(d) 0 7 0 7
sgCtrl sgSTAT1
150
100
50
Z138
STAT1
IFNAR1
IRF-1
IRF-2
IRF-7
IRF-9
TYK2
S63845(d) 0 7 0 7
sgCtrl sgSTAT1
3
2
1
D
DLBCL BL
p=7.7e-05
r=-0.26
Log2 expression (CXCL9)
Log2 expression (MYC)
p=0.00031
r=-0.24
Log2 expression (CXCL10)
Log2 expression (MYC)
p=7.1e-06
r=-0.3
Log2 expression (CXCL11)
Log2 expression (MYC)
E
DLBCL BL
p<2.2e-16
r=0.78
Log2 expression (IRF-1)
Log2 expression (STAT1)
p<2.2e-16
r=0.62
Log2 expression (IRF-7)
Log2 expression (STAT1)
p<2.2e-16
r=0.72
Log2 expression (IRF-9)
Log2 expression (STAT1)

Supplementary Figure 4. MYC downregulation induced by S63845 treatment leads to STAT1 upregulation and activation of Type I IFN signaling pathways. A. Heatmap showing upregulation of IFN and inflammatory pathway related genes in parental cell lines (HBL-2, U2932 and REC-1) treated with the IC50 dose of S63845 at different time points. **B.** Heatmap showing increasing expression of STAT1, CXCL9, CXCL10, CXCL11, and genes associated with IFN and inflammatory signaling pathway in MYC knockdown Namalwa cells compared to parental controls**.** **C.** Heatmap showing elevated levels of STAT1, CXCL9, CXCL11, and genes associated with IFN and inflammatory in Z138 parental and MYC knockdown cells treated with the IC50 dose of S63845 at different time points**. D.** Correlation plots showing inverse relationships between MYC mRNA levels and CXCL9, CXCL10 and CXCL11 mRNA levels in classical MYC-driven human lymphoma patient samples (DLBCL, orange; BL, purple). **E.** Correlation plots showing positive association between mRNA levels of STAT1 and IRF-1, IRF-7, IRF-9 mRNA level in classical MYC-driven human lymphoma patient samples (DLBCL, orange; BL, purple).

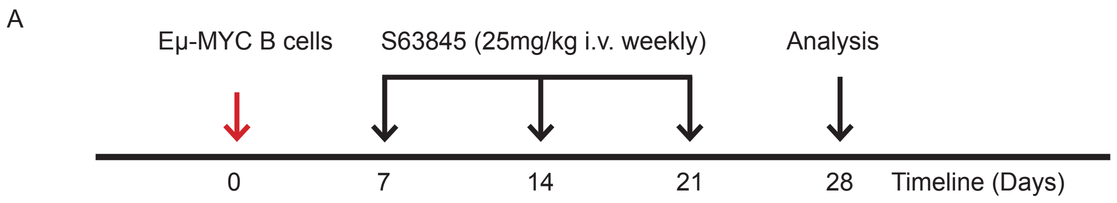

Supplementary Figure 5. MCL-1 inhibition with S63845 reinvigorates TME towards immunogenicity in a syngeneic MYC-driven lymphoma mouse model. A. Schematic of the Eμ-MYC lymphoma-bearing mouse model. Mice were injected intravenously with Eμ-MYC B cells and treated weekly with S63845 beginning on day 7 post-injection for a total of 3 treatments.

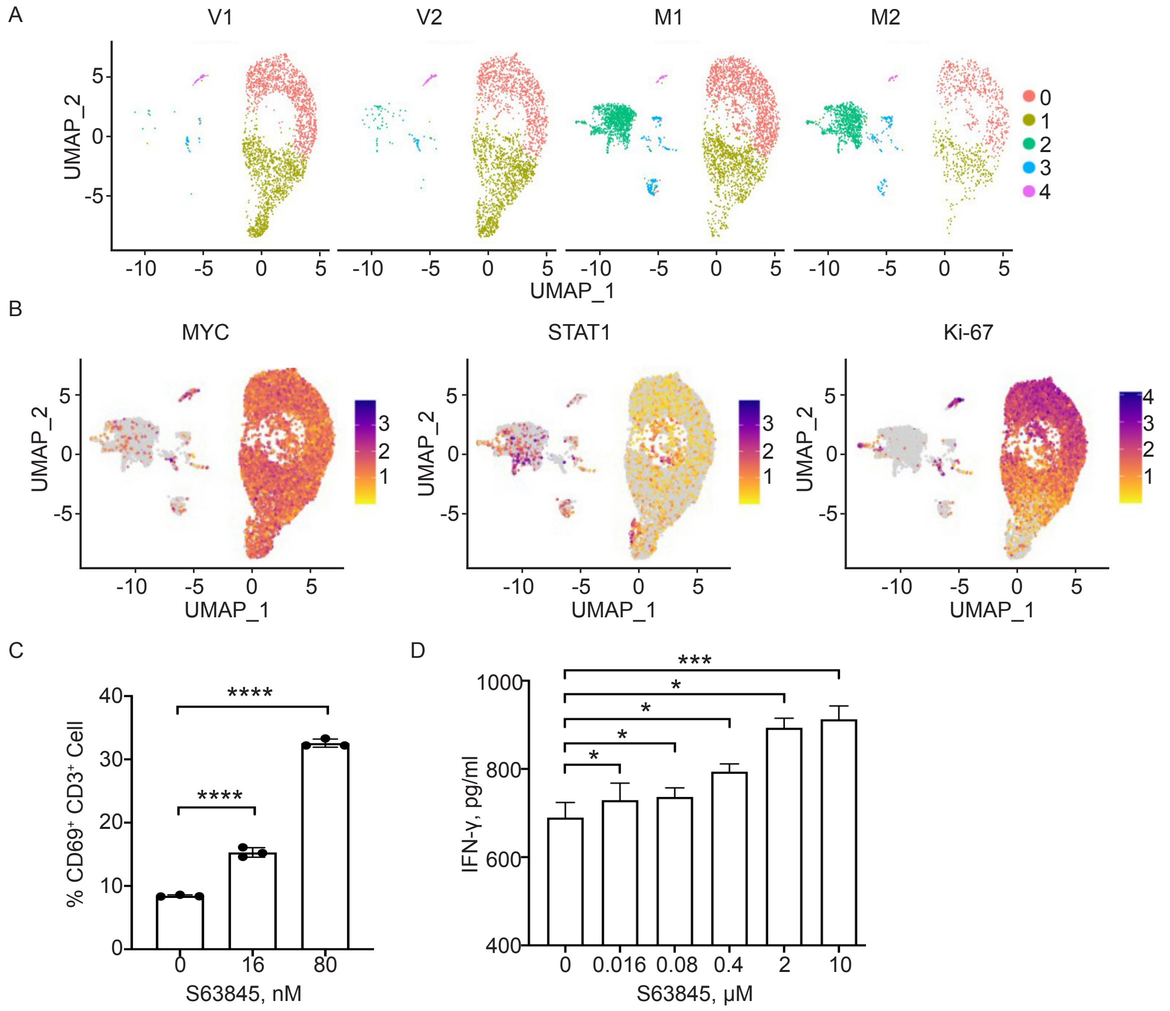

Supplementary Figure 6. Single-Cell RNA-seq reveals tumor-intrinsic and microenvironmental remodeling following MCL-1 inhibition in MYC associated B-cell lymphoma. A. scRNA-seq analysis of UMAP plot was colored by graphed-base cell clusters in untreated (V1 and V2) and MCL-1i treated (M1 and M2) tumors, respectively. **B.** UMAP plots showing the mRNA expression and distribution of MYC (left), STAT1 (middle) and Ki-67 (right) of untreated and MCL-1i treated tumors. Each cell was colored based on normalized mRNA level of indicated genes. **C.** Increased proportions of CD69+ cells among CD3+ T-cell in CD19 CAR-T cells following S63845 treatment. **D.** Interferon-γ production of CD19 CAR-T treated with S63845.

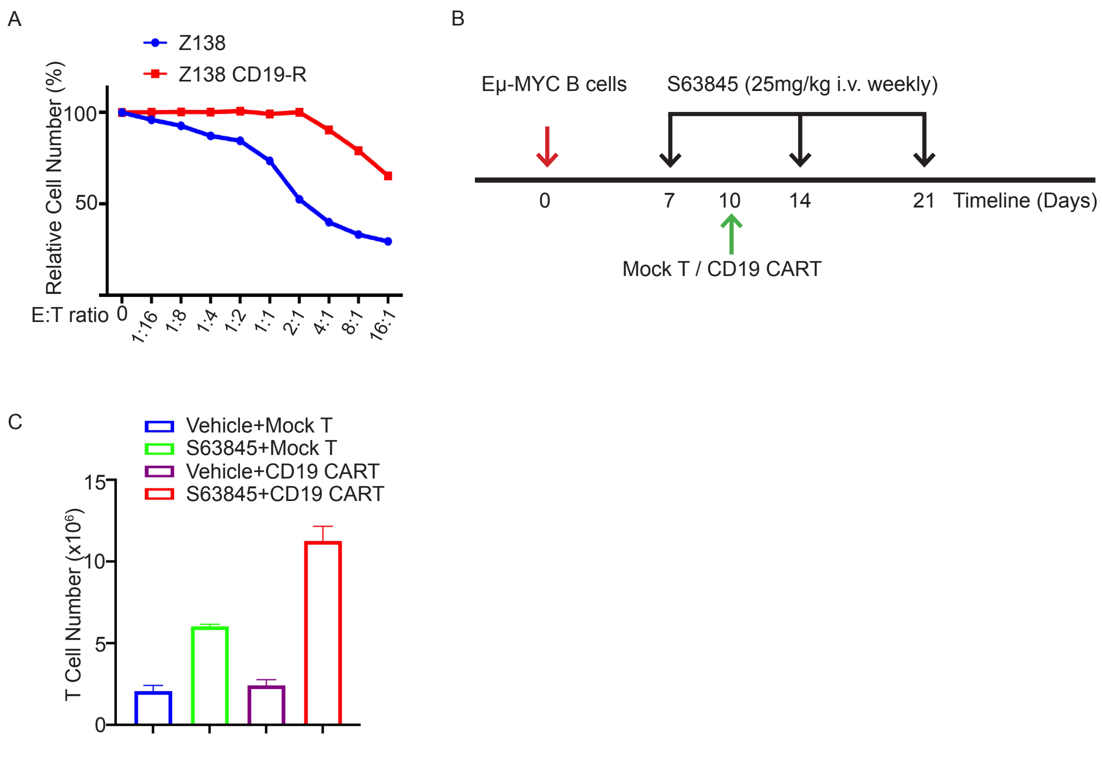

Supplementary Figure 7. Combination of MCL-1 inhibition and CD19 CAR-T therapy overcome resistance in aggressive B-cell lymphoma. A. CD19 CAR-T cytotoxicity assay in paired CD19 CAR-T sensitive parental cells and resistant lymphoma cell lines. **B.** Schematic of the Eμ-MYC lymphoma-bearing mice model. S63845 was administered intraperitoneally at a dose of 25 mg/kg once per week on days 7, 14, and 21. CD19 CAR-T or mock T cells were injected intravenously on day 10. **C.** Increased numbers of T cells in spleen.
